# The Lighting Environment, Its Metrology, and Non-visual Responses

**DOI:** 10.3389/fneur.2021.624861

**Published:** 2021-03-04

**Authors:** Luc J. M. Schlangen, Luke L. A. Price

**Affiliations:** ^1^Department Human-Technology Interaction, Intelligent Lighting Institute, Eindhoven University of Technology, Eindhoven, Netherlands; ^2^Centre for Radiation, Chemical and Environmental Hazards, Public Health England, Didcot, United Kingdom

**Keywords:** melanopsin, intrinsically-photosensitive retinal ganglion cells, circadian rhtyms, melatonin, visual perception, non-image forming effects of light, sleep, light therapy

## Abstract

International standard CIE S 026:2018 provides lighting professionals and field researchers in chronobiology with a method to characterize light exposures with respect to non-visual photoreception and responses. This standard defines five spectral sensitivity functions that describe optical radiation for its ability to stimulate each of the five α-opic retinal photoreceptor classes that contribute to the non-visual effects of light in humans via intrinsically-photosensitive retinal ganglion cells (ipRGCs). The CIE also recently published an open-access α-opic toolbox that calculates all the quantities and ratios of the α-opic metrology in the photometric, radiometric and photon systems, based on either a measured (user-defined) spectrum or selected illuminants (A, D65, E, FL11, LED-B3) built into the toolbox. For a wide variety of ecologically-valid conditions, the melanopsin-based photoreception of ipRGCs has been shown to account for the spectral sensitivity of non-visual responses, from shifting the timing of nocturnal sleep and melatonin secretion to regulating steady-state pupil diameter. Recent findings continue to confirm that the photopigment melanopsin also plays a role in visual responses, and that melanopsin-based photoreception may have a significant influence on brightness perception and aspects of spatial vision. Although knowledge concerning the extent to which rods and cones interact with ipRGCs in driving non-visual effects is still growing, a CIE position statement recently used melanopic equivalent daylight (D65) illuminance in preliminary guidance on applying “proper light at the proper time” to manipulate non-visual responses. Further guidance on this approach is awaited from the participants of the 2nd International Workshop on Circadian and Neurophysiological Photometry (in Manchester, August 2019). The new α-opic metrology of CIE S 026 enables traceable measurements and a formal, quantitative specification of personal light exposures, photic interventions and lighting designs. Here, we apply this metrology to everyday light sources including a natural daylight time series, a range of LED lighting products and, using the toobox, to a smartphone display screen. This collection of examples suggests ways in which variations in the melanopic content of light over the day can be adopted in strategies that use light to support human health and well-being.

## Introduction

Light is essential for vision, but starting from the earliest weeks of life (1–5) it also drives important non-image-forming (NIF) effects that are powerful determinants of sleep (6), circadian rhythms (7), alertness (8, 9), mood (10) and hormone secretion (11). This paper is intended for lighting professionals, policy makers and researchers with a practical interest in lights' eye-mediated NIF effects, chronobiology and health. It explains and discusses a standardized light metrology (12) that is based on five retinal photoreceptor types, each of which has a distinct spectral sensitivity and may contribute to non-visual or NIF responses (13). Significantly, melanopsin is the functional photopigment for one of these five photoreceptor types.

Accumulating evidence (6, 14–21) suggests that the spectral sensitivity of melanopsin is the most successful and parsimonious model to predict responses to medium and long duration exposures to ambient light like circadian phase shifting, or modulations in pupil-size, alertness, and melatonin secretion. However, no single action spectrum or proxy will ever provide the complete picture (13, 22) for all the testable variations in intensity, timing, duration, and patterns of light exposure that can be created in laboratory settings (23, 24). Moreover, the effects of light in field settings are often confounded by various uncertainties which may be due to non-photic effects, interindividual variations in sensitivity to light (25), differences in the populations studied and the reduced environmental and behavioral control in real-life environments. Whilst acknowledging these limitations, some examples will be presented to suggest ways in which the melanopsin-based quantities from the standardized light metrology (12) can already be applied in practice.

The pineal hormone melatonin is an important, commonly used marker of circadian rhythms and the effects of light on its nocturnal secretion are well-established (11, 14, 15, 26, 27). In humans, melatonin facilitates sleep initiation and sleep consolidation (28), and is only secreted (resulting in detectable levels) during the period that we habitually sleep. Nocturnal light exposure acutely suppresses circulating melatonin levels (11), but being awake, or asleep, by itself has no direct effect on urinary melatonin (29). Under constant dim light conditions, melatonin levels start rising in the evening and peak at night about 2 h before the core body temperature reaches its nadir (denoted as CBTmin), with this nadir typically occurring a further 2 h before (habitual) wake-up time (30, 31).

The sleep-wake cycle closely follows the 24 h melatonin cycle: habitual bedtime is about 2 h after the melatonin onset (in dim light), while habitual wake-up typically occurs about 10 h after melatonin onset (in dim light), with melatonin onset being defined as the time point at which the salivary melatonin concentration increased to and stayed above either 4 pg/ml or 25% of its fitted amplitude (32, 33). Around the habitual wake-up time, melatonin concentrations are decreasing and drop to undetectable levels, even in dim light conditions. When living outdoors for a week in summer, camping under natural light and without any electric light exposure, average melatonin onset occurs near sunset, while average melatonin offset occurs before wake time, just after sunrise (34). An abrupt change of the sleep-wake cycle leaves the melatonin 24 h profile (virtually) unaffected (35), whilst a single laboratory light exposure with the appropriate timing and duration can shift the phase of the melatonin rhythm by up to 3 h (27, 36). However, negative feedback in the genetic clock mechanism, regulated by *Sik1*, limits the phase-shifting effects of light (37) and in jet-lagged humans and most other mammals behavioral phase shifts remain restricted to about 1 h per day (one time zone) (38).

**Figure 1:**
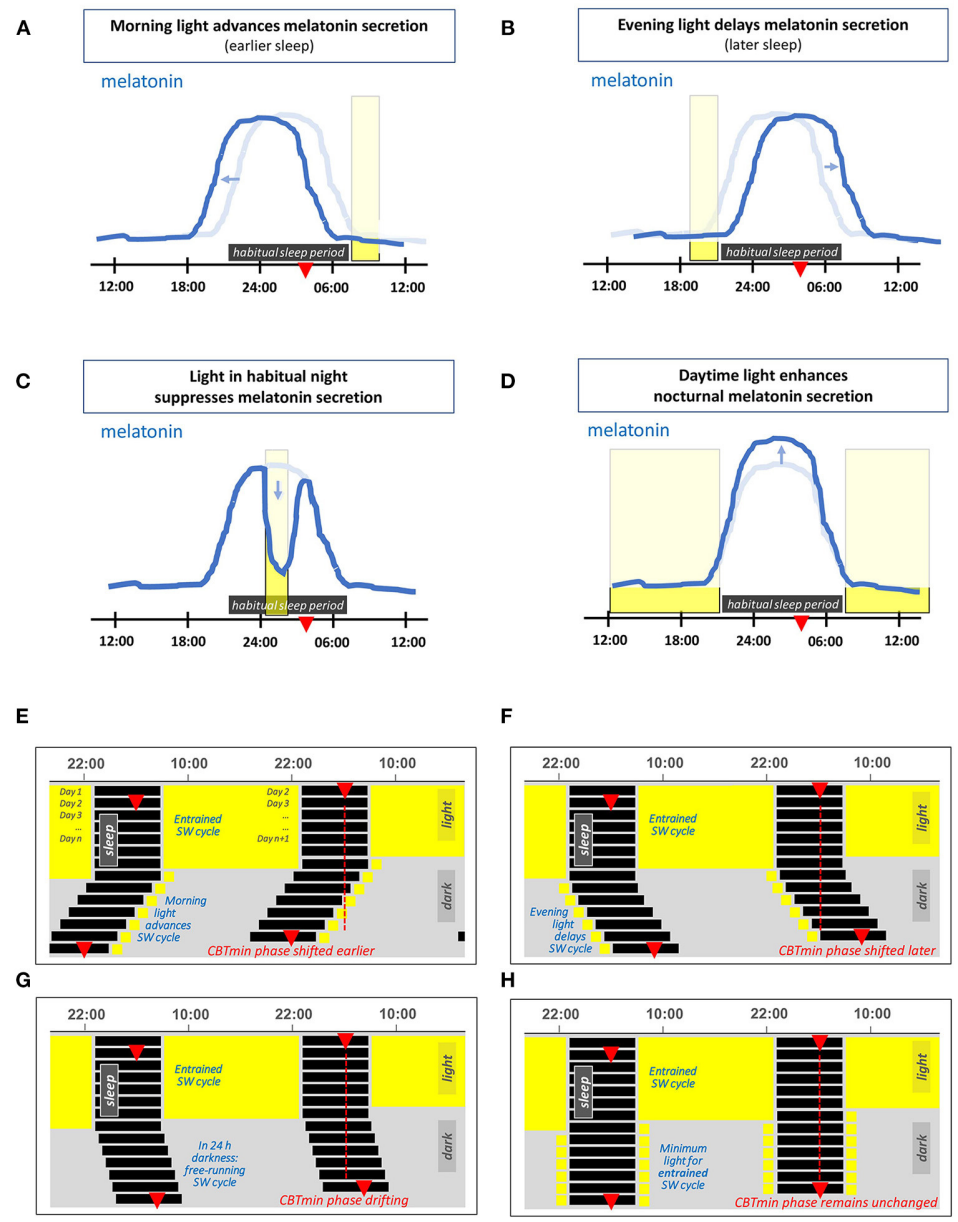
**(A–D)** Schematic representation of the effects of light on the 24 h melatonin profile. This profile marks the circadian rhythm and the habitual sleep period. The latter is indicated by the horizontal dark rectangle, the light blue line represents the corresponding melatonin profile for an individual in 24 h dim light conditions. The red triangle indicates the time at which the core body temperature reaches its nadir at about 2 h before (habitual) wake-up time. The vertical rectangles denote a particular light exposure. **(A)** Light exposure in the morning advances the timing of melatonin secretion (i.e., supports earlier bedtime and awakening). **(B)** Light exposure in the evening delays the timing of melatonin secretion. **(C)** Light exposure during the habitual sleep period acutely suppresses melatonin secretion. **(D)** Daytime light exposure strengthens subsequent nocturnal melatonin secretion. **(E–H)** Double-plotted actograms schematically showing patterns of the human sleep-wake (SW) cycle resulting from different light exposures, each starting with several days in 16L:8D and with light restricted on subsequent days. **(E)** Light restricted to 1 h in the morning on waking, **(F)** light restricted to 1 h in the late evening light. **(G)** In complete darkness (a D:D cycle), since the intrinsic period of the circadian rhythm in humans slightly exceeds 24 h, the timing of the SW cycle drifts later and later across days. **(H)** A theoretical example with sufficient light each morning and evening to entrain the SW cycle.

The effects of light on the 24 h melatonin profile are shown schematically in Figures 1A–D. Morning light exposure advances the timing of melatonin secretion, facilitating earlier bedtimes and sleep onset, while evening light exposure postpones melatonin secretion, thus delaying the drive to go to bed (27). The circadian system considers light exposure that occurs before the CBTmin to be evening light, whereas light exposure that occurs during the hours after the CBTmin is considered to be morning light (27). Daytime light exposure can enhance nocturnal melatonin secretion (39), strengthen the body clock and reduce sensitivity to late evening/nighttime light exposures (40–45). Even a single 2.5 h bright light exposure in the early evening is sufficient to reduce the acute sleep-disruptive effects of late evening light exposure (46).

Figures 1E,F show the effect of morning and evening light on the sleep-wake cycle within a double-plotted actogram. When the light-dark cycle has a low amplitude, i.e., insufficient contrast between day and night, the circadian rhythm is free-running. A person that lives in constant dim light, has a sleep-wake cycle that shifts slowly to a later time every next day. This is depicted in Figure 1G, and is due to the fact that under dim light the circadian rhythm is free running at its endogenous period, which is on average about 24.2 h for humans (35, 47–50). The right combination of morning and evening light exposure entrains the circadian rhythm, so that it remains in sync with the 24 h light-dark cycle, see Figure 1H.

Evidence from the US suggests people in modern society may spend around 90% of their time indoors (51–53). The typical human indoor environment provides relatively little light during daytime, especially compared to the natural light outdoors, where illuminances may be 1, 2, or even 3 orders of magnitude higher. For instance, the European standard for lighting of work places (54) specifies minimum values for maintained horizontal illuminance in offices between 200 and 750 lx, depending on the specific task, whereas the horizontal illuminance outdoors can be as high as 150 klx (55). In the late-evening hours and at night, the widespread use of electrical light and luminous display devices results in extended exposures to light (56). Through their impact on circadian rhythms, these unnatural lighting conditions enhance eveningness (34). Moreover, modern lifestyles and (unnatural) light exposures are known to result in more “social jet-lag,” and this has negative consequences for sleep, performance, well-being and health (57, 58). Evolution shaped us to live in much brighter daytime conditions than present in our modern indoor life. For a healthy lit environment, people with a normal diurnal activity pattern (i.e., day-oriented, and usually in bed at night) need bright white light during the day, and especially in the morning, while they should reduce prolonged exposures in the late evening and avoid light as much as possible at night [see also CIE position statement (59)].

Although the introduction concentrates on chronobiology, it should be noted that chronobiological responses are just a subset of non-visual responses to light. The non-visual metrology tools described in this paper, and the information presented below, can also be applied to other retinal responses to ambient light.

## Retinal Photoreceptors

**Figure 2:**
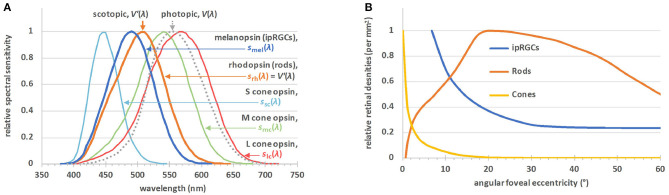
**(A)** The α-opic action spectra for non-visual metrology (12), *s*_α_(λ); S-cone opic (α = sc), M-cone-opic (α = mc), L-cone opic (α = lc), rhodopic (α = rh), or melanopic (α = mel), where *s*_rh_(λ) is defined to be equal to the spectral luminous efficiency function for scotopic vision, *V*′(λ). The spectral luminous efficiency function for photopic vision, *V*(λ), is also plotted. **(B)** The relative densities of the rods, cones and ipRGCs by angular eccentricity from the central fovea (61, 62). There are no ipRGCs in the central visual field, but outside this field their density falls off to a steady value. The maximum density of the ipRGCs is ~20–25 cells·mm^−2^, 4 orders of magnitude lower than the maximum densities of the rods or cones.

Early this century a new class of retinal photoreceptor, the intrinsically-photosensitive retinal ganglion cell (ipRGC), was discovered (60). In addition to receiving extrinsic input signals from rods and cones, this class of photoreceptor expresses melanopsin which gives rise to the intrinsic light sensitivity after which it is named (13). Figure 2A shows the spectral sensitivities of the five classes of photoreceptors involved in non-visual photoreception (12), together with the well-known *V*(λ) function officially denoted as *the spectral luminous efficiency function for photopic vision*. In humans, melanopsin photoreception occurs efficiently across the short wavelength range of the visible spectrum between 420 and 560 nm, with a peak sensitivity *in vivo* at ~490 nm (13). Melanopsin-based signaling is more sluggish in onset and more sustained than rod or cone signaling (63–65). At least six subtypes of ipRGCs, M1–M6, have been identified in the mammalian retina (M1–M5 to date in humans) (66–69). Unlike rods and cones, ipRGCs have photosensitive dendrites that extend transversely across the retina. Figure 2B shows the relative densities of the rods, cones and ipRGCs as a function of retinal eccentricity. Melanopsin-based photoreception predicts both clock-mediated and acute non-visual responses under a range of everyday light exposures (21). The clock-mediated effects include regulation of the sleep-wake cycle and circadian phase shifting, whereas melatonin suppression, control of alertness and the steady state pupil diameter are examples of acute responses to light (17, 18, 20, 21).

During the first 5 years after birth, the crystalline lens in the human eye is still transmissive for short wavelength visible light and even for ultraviolet radiation (UVR) close to 320 nm (70). It becomes opaque to UVR at about an age of 5, and as age increases, the lens transmittance in the short wavelength range (i.e., violet and blue) of the visible spectrum decreases. Consequently, retinal photoreceptors receive less light input at older ages, particularly the short-wavelength sensitive photoreceptors (rods, S-cones and ipRGCs). Although adaptation mechanisms and neural plasticity may compensate for the age-induced decline in short-wavelength light that actually reaches the retina, the number of ipRGCs drops with age advancing beyond 50 (71). This loss of ipRGCs is accompanied by changes in cell morphology and an observable increase in randomness of the ipRGC distribution pattern.

It has been suggested that a decline in melanopsin photoreception with age could play a significant, deteriorating role in sleep and neuro-cognitive effects of aging (71), including those related to dementia as well as general senescence. It is plausible that these effects may be partly mediated by the negative effects on sleep due to a compromised non-visual circadian regulation with increasing age (72–74). Partly corroborating this hypothesis, it has also been observed that more fragmented and less stable sleep-activity patterns are associated with a higher all-cause mortality (up to ~20%) in the middle-aged and the elderly, independently of age (75).

## Quantifying Light for Luminous Perception

Traditional lighting practice primarily targets visual performance, comfort and other aspects of the visual domain, quantifying lighting designs and installations and light exposures using luminous flux (in lumens), illuminance (in lux) and other visually related quantities. These quantities describe the luminous sensation of a light source under photopic conditions [i.e., for luminances above 5 cd/m^2^ (76)], where cones drive human visual responses. Scotopic vision occurs while the eye is adapted to very low luminances (below 0.001 cd/m^2^). Under scotopic conditions, visual responses are driven by rods. The conversion between luminance and illuminance depends on the apparent source size measured in steradians, so general scotopic and photopic thresholds cannot be expressed in lux.

Individually, photoreceptors follow the principle of univariance, meaning they cannot discriminate between a change in intensity and a change in wavelength (77). As such, the spectral sensitivities of the human luminous sensation for photopic and scotopic vision can be described by the spectral luminous efficiency functions *V*(λ) and *V*′(λ), respectively, see Figure 2A. The spectral power of light, for instance, can be photopically-weighted or scotopically-weighted by multiplying each wavelength by *V*(λ) or *V*′(λ), respectively. Photometric units (such as the lumen, lux or candela) are obtained after summing the result (which is now a photopically- or scotopically-weighted spectrum) over all wavelengths and multiplying the result by the corresponding efficacy constants (*K*_m_ and *K*′_m_, respectively), as described below.

By definition, monochromatic radiation with a frequency of 540 × 10^12^ Hz, (which corresponds to the wavelength 555 nm in standard air^1^) has a luminous efficacy of 683 lm/W (78). Since the *V*(λ) function reaches its peak value at 555 nm, this is where the maximum luminous efficiency for photopic vision (denoted by constant *K*_m_) equals 683 lm/W. The maximum luminous efficiency for scotopic vision (denoted by constant *K*′_m_) equals 1,700 lm/W, which follows from the relationship *K*_m_·*V*(555 nm) = *K*′_m_·*V*′(555 nm).

The ratio of the luminous output (of a source) as evaluated using the scotopic efficiency function to the luminous output evaluated using the photopic efficiency function is known as the S/P ratio_._ The S/P ratio is a characteristic of the spectral distribution of the light, and by definition, equals 1 for monochromatic radiation with a frequency of 540 × 10^12^ Hz, or a wavelength of 555 nm (in air). An S/P ratio above 1 denotes that a light source is more activating to rods per (photopic) lumen than 1 lumen of monochromatic light at 555 nm.

Mesopic vision occurs while the eye is adapted to light levels that are in between photopic and scotopic conditions. In this range, i.e., in the mesopic regime, the combined action of rods and cones defines the human visual response. However, ipRGCs are implicated in retinal adaptation (79) and may be involved in the regulation of mesopic and photopic visual sensitivity (80).

Do and Yau (81) provided an extensive review of ipRGCs and their functions, including their roles in visual responses. Already in 2002, Hankins and Lucas had demonstrated that adaptations of the human primary cone visual pathway according to time of day are driven by a non-rod, non-cone photopigment with a spectral sensitivity profile that matches the standard profile of an opsin:vitamin A-based pigment with a peak at ~483 nm (79). The resulting curve is now widely accepted as the prototype action spectrum of the photopigment melanopsin and describes the intrinsic light sensitivity in ipRGCs. Another demonstration that melanopsin can drive visual perception comes from a case study of a blind individual lacking functional rods and cones who could report whether a monochromatic light stimulus of 480 nm was on or off, but failed to do so for other wavelengths (82).

Recent studies suggest the possibility of further melanopic influences on visual responses. Human brightness perception can be greater when the light stimulus has a larger melanopic content while being isoluminant for rods and cones (83), and further experiments have quantified the effect of melanopsin on brightness perception in more detail (84, 85). Melanopsin effects can increase brightness perception by up to 10%, especially for brightness discrimination tasks that involve little or no differences in luminance and hue (86). Finally, it is worth noting that melanopsin photoreception can also improve the detectability of coarse patterns (80). Together these results indicate that melanopsin is not only implicated in non-visual responses and visual adaptation, but may also contribute meaningfully to further visual responses like brightness perception and pattern recognition. However, proper demonstration of melanopic influences to vision is methodologically complex and still faces many challenges (87). At present, the relevance of melanopsin-based photoreception for brightness perception beyond laboratory settings is not yet settled and merits further investigation.

## Quantifying Light for Non-Visual Responses: α-OPIC Metrology

As detailed above, the melanopsin-based photoreception of ipRGCs constitutes an important driver of non-visual responses. In their work, many lighting designers already draw on a wide understanding of the visual, architectural and psychological aspects of light and lighting. Awareness amongst lighting professionals is increasing that next to cone-dominated metrics such as correlated color temperature (CCT), illuminance and luminance, there is a need to consider melanopsin-based photoreception in specifications, codes, recommendations and research. All these metrics are useful tools for quantifying or comparing individual aspects within a lighting scheme, but they cannot replace an experienced designer's overall appreciation of the interplay between the diverse effects of light. In addition, NIF photoreception relates to the light arriving at the eyes from all directions. This requires recommendations framed in terms of light arriving at eye level—e.g., measured normal to the visual axis in the vertical plane—rather than with reference to the light falling on the horizontal plane, walls or object surfaces.

No single action spectrum or proxy can describe all eye-mediated non-visual responses to light (13, 22). All five known receptor types can contribute to these responses, and the relative contribution of each individual photoreceptor type can vary depending on the specific response and upon light exposure properties such as intensity, spectrum, duration, timing (external and internal/circadian), prior light history and sleep deprivation state of the individual. Based on the Lucas et al. review paper (13), the International Commission on Illumination (CIE)—the worldwide body responsible for developing international standards and reports on light and lighting—has published CIE S 026:2018 “*CIE System for Metrology of Optical Radiation for ipRGC-Influenced Responses to Light*” (12). This new International Standard defines spectral sensitivity functions, quantities and metrics to describe optical radiation for its ability to stimulate each of the five retinal photoreceptor classes that, via ipRGCs, can contribute to the non-visual effects and functions of light in humans.

The Lucas et al. (13) authors used an opsin template and a lens transmittance function to establish five action spectra that describe the spectral sensitivity of all five known retinal photoreceptors that can contribute to non-visual responses. The CIE standard (12) adopts the same melanopsin action spectrum as the Lucas et al. (13) authors. However, for consistency with existing standards and psychophysical data, CIE S 026 adopts the 10-degree cone fundamentals (88) and the spectral luminous efficiency function for scotopic vision, *V*'(λ), to describe the cone and rod action spectra, respectively.

Figure 2A shows the five spectral weighting functions or action spectra, *s*_α_(λ), for the five retinal photoreceptor classes: S cone, M cone, L cone, rhodopsin and melanopsin-encoded photoreception of ipRGCs as defined in CIE S 026. For each of these five (α-opic) photoreceptors, an α-opic irradiance can be calculated from the spectral irradiance, *E*_e,λ_, of a (test) light source, see Table 1. The α-opic irradiance of a test light divided by its illuminance, *E*_v_, defines its α-opic efficacy of luminous radiation (α-opic ELR). The ratio of this α-opic ELR to the α-opic ELR of standard daylight (D65) defines the α-opic daylight (D65) efficacy ratio (α-opic DER) of the test light.

**Table 1:** Glossary of α-opic metrology (12), where *s*_α_(λ) refer to the α-opic action spectra shown in Figure 2A, *K*_α,*v*_ is the “α-opic stimulus per lumen,” *K*_α,*v*_ calculated for D65 (i.e., the α-opic ELR for D65, $K_{\alpha ,v}^{\text{D}65}$) is a normalization constant. There are two ways to calculate the α-opic DER: α-opic DER = α-opic ELR / α-opic ELR for D65 = α-opic EDI / illuminance.

**Quantity**	**Abbreviation**	**Formula**	**Meaning**	**Unit**
α-opic irradiance *see note 1*	–	*E*_α_ = ∫*E*_*e*, λ_(λ)*s*_α_(λ)*dλ*	Weighted spectral irradiance, *E*_*e*, λ_, integrated over wavelength	W·m^−2^
α-opic efficacy of luminous radiation	α-opic ELR	*K*_α, *v*_ = *E*_α_*E*_*v*_	Quotient of α-opic irradiance, *E*_α_, and illuminance, *E*_*v*_	W·lm^−1^
α-opic equivalent daylight (D65) illuminance	α-opic EDI	$E_{v,\alpha }^{D65}=E_{\alpha }K_{\alpha ,v}^{D65}$*see notes 2 & 3*	Illuminance level of daylight (D65), producing an equal α-opic irradiance, *E*_α_, as the test light	lx
α-opic daylight (D65) efficacy ratio	α-opic DER	$\gamma_{\alpha ,v}^{D65}=K_{\alpha ,v}K_{\alpha ,v}^{D65}$*see note 2*	Ratio of the α-opic ELR of the test light, *K*_α, *v*_, to the α-opic ELR of daylight (D65), $K_{\alpha ,v}^{D65}$	–

*Note 1. The α-opic photoreceptor types are denoted by five subscripts: S-cone opic (α = sc), M-cone-opic (α = mc), L-cone opic (α = lc), rhodopic (α = rh) and melanopic (α = mel), and the spectral weighting functions *s*_α_(λ) are shown in Figure 2A*.

*Note 2. The five normalization constants, $K_{\alpha ,v}^{\text{D65}}$, are $K_{sc,v}^{\text{D65}}$ = 0.8173 mW·lm^−1^, $K_{mc,v}^{\text{D65}}$ = 1.4558 mW·lm^−1^, $K_{lc,v}^{\text{D65}}$ = 1.6289 mW·lm^−1^, $K_{rh,v}^{\text{D65}}$ = 1.4497 mW·lm^−1^, and $K_{mel,v}^{D65}$ = 1.3262 mW·lm^−1^*.

*Note 3. The non-standard quantity “melanopic equivalent illuminance” (often referred to as EML) can be converted into the melanopic EDI by a multiplication with 0.9058 (i.e., the α-opic DER of illuminant E). The other “α-opic equivalent illuminances” have no such linear relationship with their α-opic EDI analogs, as CIE S 026 and Lucas et al. (13) use slightly different action spectra for the rods and cones*.

## Reference Illuminants, Equivalent Illuminances, S/P, and M/P Ratios

Since daylight is a naturally occurring stimulus under which we evolved, it is an interesting and relevant point of reference to evaluate and express the properties of human light conditions within the built environment. The CIE standard illuminant D65 is adopted as the reference illuminant in CIE S 026 (2018) to express each of the five α-opic irradiances as a photometric equivalent quantity^2^. These quantities are the five α-opic equivalent daylight (D65) illuminances (α-opic EDIs). Each α-opic EDI is expressed in lx and corresponds to the illuminance of D65 radiation that is required to provide an equal α-opic irradiance as the test light, for a given α-opic photoreceptor. The term “test light” used here refers to the light being considered, to differentiate it from the reference illuminant.

The photometric equivalent concept adopted in S 026 is not restricted to illuminance (unit lx), and luminance (unit cd/m^2^). It can also be applied to other quantities such as light exposure (unit lx·h), luminous energy (unit lm·s), and luminous intensity (unit cd) ^3^.

Returning to CIE S 026, when describing the spectral properties of a test light, the ratio of the α-opic EDI of a test light to its illuminance defines the α-opic DER of the test light, see Table 1. In other words, the melanopic DER represents the ratio of the melanopic flux (“M”) per photopic luminous flux (“P”) of a test light, and this dimensionless quantity can usefully be thought of as the new “M/P ratio.” By definition, this ratio is normalized to 1 for the reference illuminant D65. The S/P ratio is an established lighting metric. It equals 1 for monochromatic radiation of 555 nm, as the S/P ratio effectively uses radiation of 555 nm as its normalizing reference illuminant. In case the melanopic EDI is 30 lx, the test light has the same activating effect on ipRGCs as 30 lx of radiation conforming to the spectrum of D65 daylight. In the same way, a scotopic illuminance of 30 lx indicates that the test light has the same effect on rods as 30 lx of radiation at 555 nm.

## Photometric and Radiometric α-OPIC Quantities

There are three different mainstream metrological approaches for quantifying visible optical radiation:

- radiometry based on spectral energy,
- radiometry based on spectral count of photons, and
- photometry based on spectral luminous efficiency function for photopic vision, *V*(λ), and the efficacy constant, *K*_m_ (or *V*′(λ) and $K_{\text{m}}^{\prime }$ for scotopic vision).

In the SI system, radiometry is described as “the field of metrology related to the physical measurement of the properties of electromagnetic radiation, including visible light.” Radiometric quantities can be unweighted, but photobiological quantities are typically weighted according to a suitable action spectrum that describes the relative efficiency of radiation as a function of wavelength in producing an effect.

Energy-based radiometry is often used by physicists, whereas photobiologists and photochemists often use the photon system, and the light and lighting professions have a strong preference for photometry. Photometry uses special SI units like cd, lm and lx. Radiometry and photometry and their units are closely related through the current definition of the SI base constant *K*_cd_ (*K*_cd_ ≈ *K*_m_, see earlier) and the corresponding SI base unit for the photometric quantity luminous intensity, namely the candela. Of the seven SI base units (and their defining constants) the candela and its defining constant *K*_cd_ are unique in relating to human vision, rather than a fundamental physical phenomenon. The photon system is very similar to the radiometric system with energy units replaced by number of photons (requiring an adjustment^4^ to spectral weighting functions and quantities), and is often expressed after taking logs, due to the very large numbers involved.

**Figure 3:**
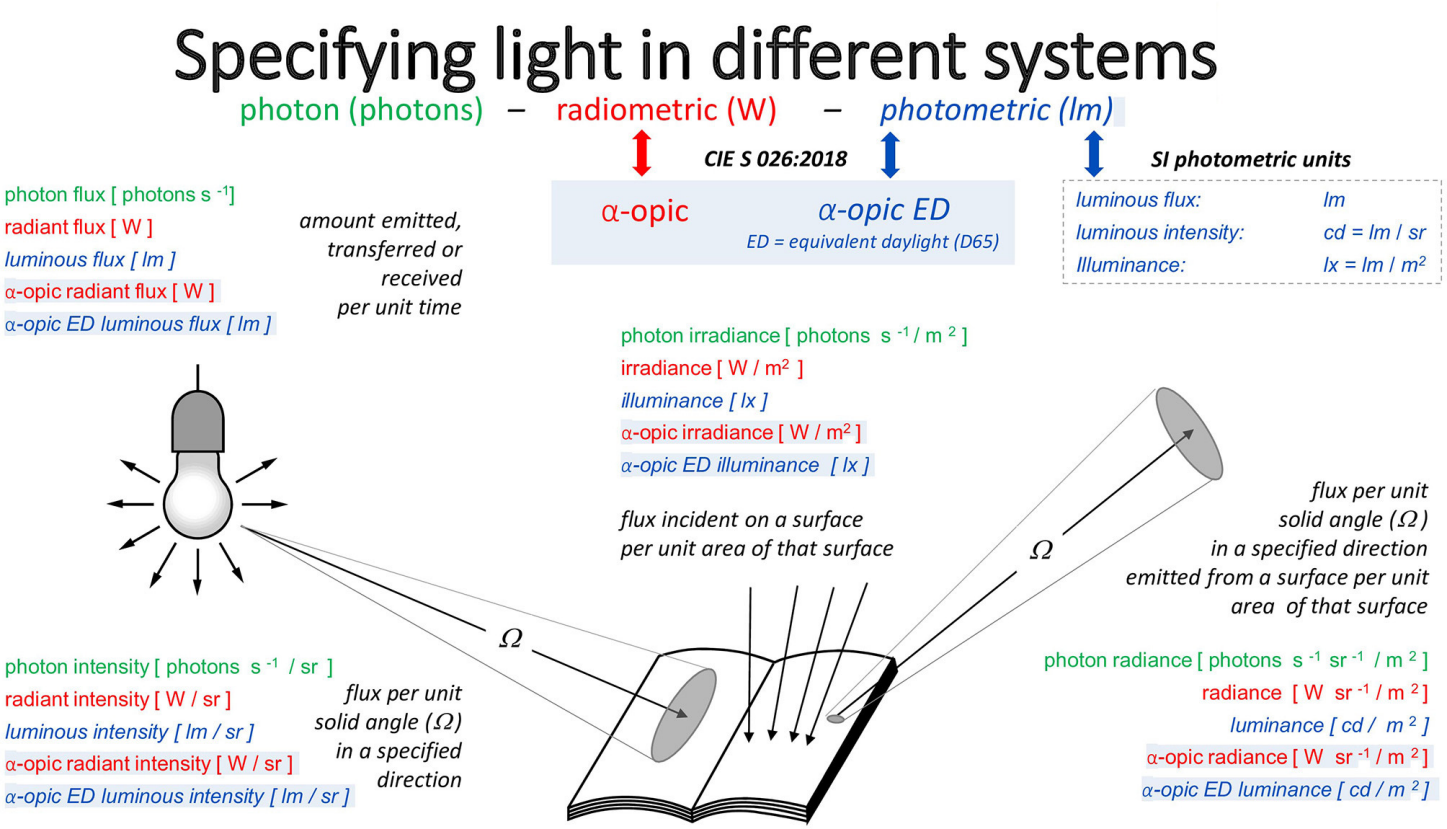
The three approaches to metrology and the α-opic quantities corresponding to these approaches.

Figure 3 illustrates the deep connections between these three metrological approaches. The set of quantities (illuminance, luminous flux, luminance, etc.) in the photometric system has the analogs *photopically-weighted* (irradiance, radiant flux, radiance) in the radiometric system and the analogs *photopically-weighted photon* (irradiance, flux, radiance) in the photon system. These analogs have units (lx, lm, cd/m^2^), (W/m^2^, W, W/sr/m^2^), and (m^−2^·s^−1^, s^−1^, sr^−1^·m^−2^·s^−1^), respectively. For melanopic quantities—with exactly the same units—the respective quantities are [melanopic EDI, melanopic equivalent daylight (D65) luminous flux, melanopic equivalent daylight (D65) luminance], *melanopic* (irradiance, radiant flux, radiance) and *melanopic photon* (irradiance, flux, radiance). Equally, for the other four α-opic quantities, the same relationships hold. Under CIE S 026 definitions, melanopic equivalent daylight (D65) luminance can be abbreviated to melanopic EDL.

## α-OPIC Toolbox

To calculate α-opic quantities in the radiometric, photon and photometric systems, and convert from one system to another, CIE has published an interactive Excel^TM^ spreadsheet, the “CIE S 026 Toolbox” (90). Access to the toolbox is free on the CIE website [doi: 10.25039/S026.2018.TB], and also an introductory video and a user guide are provided. The toolbox features include weighting functions, spectral weighting charts and a concise glossary.

**Figure 4:**
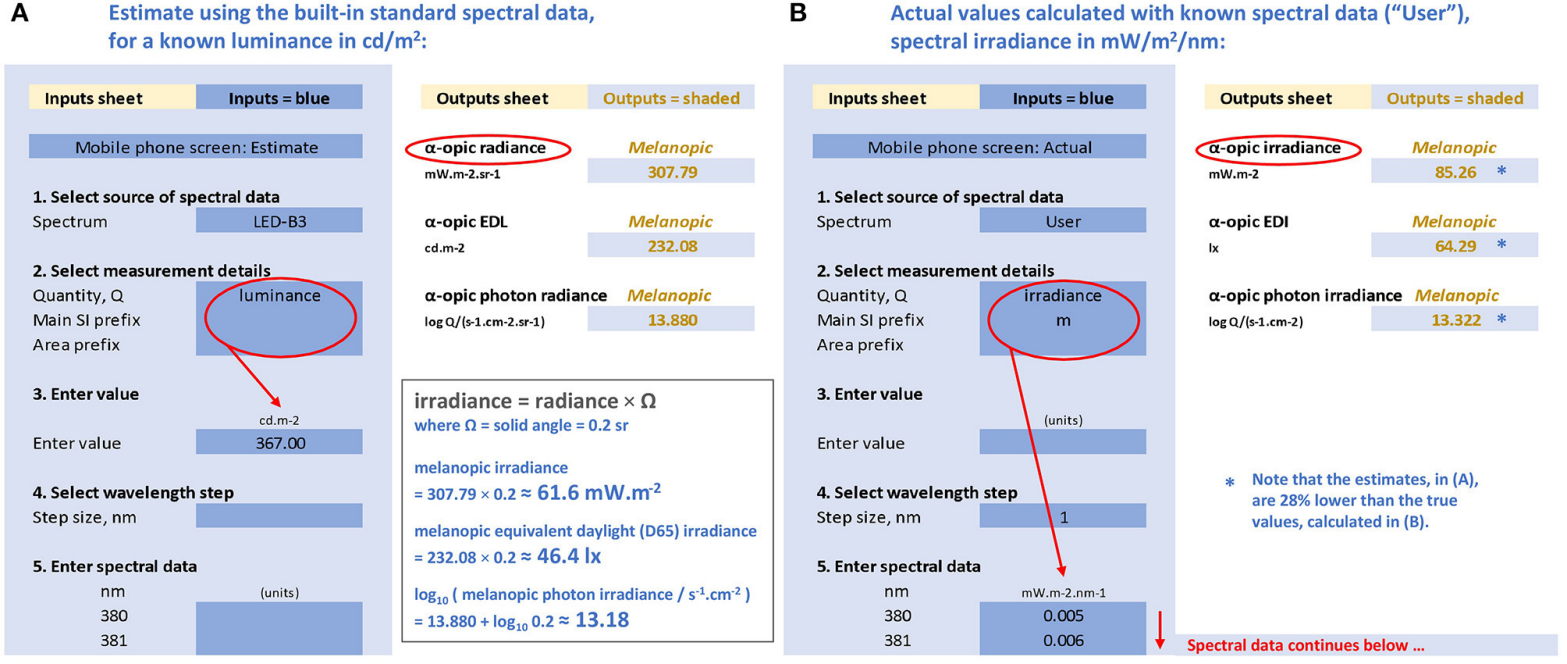
Representations of the CIE S 026 Toolbox: “Inputs” (areas with blue background) and “Outputs” (areas with white background) with numbers relating to the melanopic quantities for a mobile phone with a white screen at maximum brightness and an observer at 150 mm from the screen. **(A)** Based on the known luminance of 367 cd/m^2^, assuming the emitted spectrum conforms to CIE illuminant LED-B3, and **(B)** based on the actual measured spectral irradiance (91) with the same luminance.

Toolbox users can enter a spectral measurement and calculate all the quantities that are the geometric analogs of irradiance and radiance, including the illuminance and α-opic EDIs for this spectrum (Figure 4A). Alternatively, even without spectral data, users can familiarize themselves with the links between the three systems using one of the five built-in spectral distributions selected from the CIE standard illuminants (A, D65, E, FL11, LED-B3; Figure 4B).

## Everyday Examples

The CIE has proposed “integrative lighting” to be the official term for lighting that is specifically intended to integrate visual and non-visual effects, producing physiological and psychological effects on humans that are reflected in scientific evidence (59, 92). In the context of this promising new approach, we reconsider the light that people are exposed to in their daily lives. To investigate and characterize potential light exposures in relation to non-visual responses, a number of measurements of familiar sources of light were made, where possible re-using information from previous investigations.

The α-opic toolbox was used to evaluate the absolute and relative melanopic content of these sources in more detail. Taken together, subject to the potential limitations of the melanopic model for predicting NIF responses to light (see Introduction), the information provides useful context and further evidence for advice relating to light and health.

### Experimental Methods

All the spectral data were measured using equipment sets subject to secondary calibrations, and traceable to national standards performed, and maintained in-house (Public Health England, Didcot, Oxfordshire, UK). The data were checked against comparable alternative measurements of the same sources. Spectral equipment sets consisted of TE-cooled spectroradiometers (BW Tek, Newark, USA), coupled via optical fibers (Newport Spectra-Physics Ltd., Didcot, UK) to optical diffusers (Bentham, Reading, UK).

Daylight characteristics analyzed relate to a clear day (29 May 2020) and a cloudy day (18 June 2020), and are based on global spectral irradiance data from a solar monitoring laboratory at (51.575° N, 1.318° W, altitude 125 m), measured in the horizontal plane at 5-min intervals using in-house acquisition software (Public Health England, Didcot, Oxfordshire, UK).

**Figure 5:**
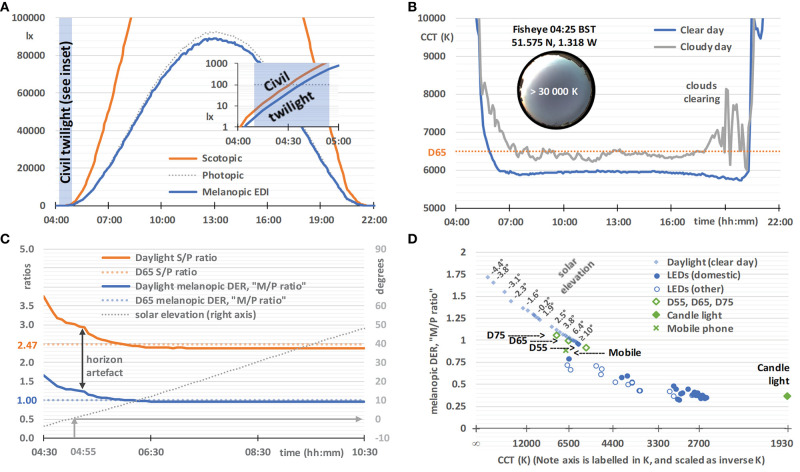
Daylight characteristics on a clear day (29 May 2020, sunrise 04:55, sunset 21:10, times in BST), based on horizontal global spectral irradiance data from a solar monitoring laboratory in Didcot, Oxfordshire, UK. **(A)** Illuminance (dotted gray line), scotopic illuminance (orange line), and melanopic EDI (blue line), with an inset semi-log graph spanning the end of twilight (04:10) and sunrise. **(B)** The correlated color temperature (CCT) on a clear day (blue line, as above from 29 May 2020) and a cloudy day (gray line, 18 June 2020, sunrise 04:50, sunset 21:25), with an inset 2π-fisheye image of the sky at 04:25. **(C)** The melanopic daylight (D65) efficacy ratio (melanopic DER, or M/P ratio, blue line), S/P ratio (orange line), and solar elevation (dotted gray line), with the melanopic DER and S/P ratio for standard daylight illuminant D65 for comparison (colored dotted lines). The vertical arrow indicates artifacts likely to be due to the slightly elevated local horizon. **(D)** Melanopic DER, or M/P ratio, plotted against an inverse-CCT axis (labeled by CCT), with solar elevation labels next to selected pairs of data points. Also plotted are CIE standard daylight illuminants (D55, D65, and D75; open diamonds), candle light (1,930 K blackbody; filled diamonds), a mobile phone (plus symbol, details given in Experimental Methods), domestic LEDs (filled circles, *n* = 25, details given in Experimental Methods), selected office LEDs and selected street light LEDs [combined as open circles, *n* = 16, also taken from (93)].

The photographic fisheye image taken at 04:25 on 29 May 2020 in Figure 5B is part of a parallel series, also taken at 5-min intervals, using Q24 hemispheric outdoor camera (Mobotix AG, Hauptsitz, Germany), at the same location.

LED spectral irradiance data were measured in temperature-controlled laboratory conditions in two earlier studies (91, 93): firstly, a modern mobile phone model (from 2016 but still in widespread use in 2021) displaying a white screen at full power at a distance of 150 mm [ID 13, (91)] and, secondly, an LED lighting sample which included any 40W-equivalent GU10 (spots) and any 60W-equivalent BC22 (bayonet light bulbs) general service lighting product types available to a UK retail consumer in 2015 over a 10-day period either online or through local and national stores (within an area bounded by Aylesbury, High Wycombe and Oxford). The latter sample included a number of comparator LED lighting products with different fittings, but excluded color-tunable products (93).

The simplified spectral emissions of a candle were modeled as arising from a Planckian radiator with a color temperature of ~1,930 K (94).

### Results: Daylight

On an ideal clear day, horizontal illuminance, scotopic illuminance and melanopic EDI follow smooth bell-shaped curves, and melanopic EDI values are similar to illuminance values (Figure 5A). This close agreement results from the melanopic EDI-normalization using standard daylight illuminant D65. The daylight characteristics in Figure 5 may not correspond exactly to daylight at high altitude, in different atmospheric conditions, and when measured with different fields-of-view. During the hour preceding dawn (see Figure 5A inset), and after sunset, the melanopic EDI increases, but decreases relative to the visual measure of illuminance, and vice versa after sunset. Other characteristics derived from the spectral daylight data also progress smoothly on a clear day, but Figure 5B illustrates how a cloudy day introduces volatility, exemplified here using the visual metric correlated color temperature (CCT). In contrast, on the clear day (verified with fisheye photographs such as the one shown in the Figure 5B inset), the CCT falls rapidly in the hours either side of dawn. The minimum CCT occurs ~1 h either after dawn or before sunset, with a small increase in CCT to a local maximum at approximately solar noon. Atmospheric conditions may give rise to asymmetry in the spectral characteristics on either side of solar noon.

Earlier studies have analyzed spectral and/or melanopic daylight time-series data averaged over a number of days (21, 95, 96). However, we are particularly interested in the results on a clear day and the melanopic daylight (D65) efficacy ratio, that, as explained earlier, can be thought of as an M/P ratio with similarities to the S/P ratio (see Figure 5C), both being ratios of the quantities shown in Figure 5A. In common with CCT, these ratios are highly dependent on solar elevation, and hence solar time on any given day. For solar elevations above 10° the ratios remained stable (i.e., for the main part of the day). For D65, with a CCT of ~6,500 K, the melanopic DER or M/P ratio equals 1 by definition and the S/P ratio equals 2.47. For solar elevations above 10°, the M/P and S/P ratios observed were slightly below 1 (see Figure 5C), which reflected the difference between the observed CCTs and that of D65 (see Figure 5B). When the sun is down or low in the sky, an elevated horizon can obscure the brightest part of the sky or the sun. In this way trees, buildings and the landscape can cause deviations from the smooth curve that would otherwise be observed. Figure 5D shows the CCT dependence of the melanopic DER for daylight on a clear day. In the next section we will compare this to white LED lighting.

### Results: White LED Lighting

Figure 5D shows the CCT dependence of the melanopic DER for the non-color-tunable white LED lighting (2015 retail products), all of which were based on a blue LED plus yellow phosphor, with the GU10 and BC22 domestic LEDs shown as a separate series. For the domestic LEDs (*n* = 25), CCT explained 87% of the variance in melanopic DER, and CCT plus CRI (Color Rendering Index, *R*_a_) explained 95% (multiple linear regression). This chart shows that this CCT dependence of the melanopic DER for the LED technology common to this white LED lighting sample does not match the CCT dependence of the melanopic DER for daylight on a clear day. Further, all the LED lighting in Figure 5D has a significantly lower melanopic DER than daylight on a clear day, typically by around 25% for a CCT of 6,500 K. At other CCT values the deficit in melanopic DER relative to daylight is higher, and it remains significant, even after adjusting for the CCT-dependencies within the daylight and LED melanopic DER series. In other words, this supports the viewpoint that all the LED lights in this sample were relatively inefficient at producing melanopic light for a given combination of CCT and luminous flux. The lower melanopic efficiency of white LED lighting with respect to natural daylight has also been reported previously (97, 98). In addition to a reduced illuminance, a lower melanopic DER may be appropriate at night and within spaces designed to be restful, whereas in active workplaces a higher melanopic DER and an elevated illuminance may engender a healthier daytime environment.

### Results: Mobile Phone Screen—Toolbox Example

To further illustrate the α-opic metrology and the S 026 Toolbox, we will consider the melanopic EDI (in lx) produced by a typical modern mobile phone (plotted as a green cross in Figure 5D). There is some concern about the effects on sleep of using display screen equipment before bedtime, including the use of mobile phones and tablets in bed, because of the light they emit (45, 99, 100), so the data we present here will provide a relevant and helpful example to place the α-opic quantities in context. Indeed, a number of groups have directly studied the effects that different light exposures can have on sleep (25, 26, 100).

There are two approaches for performing calculations available in the toolbox. The first is a simplified approach using the spectra from the five built-in standard illuminants (A, D65, E, FL11, LED-B3). The second approach requires the user to enter the actual spectral data of the test light in consideration. These two approaches are chosen to illustrate why using the simplified approach (i.e., generalizing results from standardized spectral distributions) will not always be appropriate, and may cause errors.

#### Simplified Approach

For a white mobile phone screen at full power backlit with an LED, the luminance is 367 cd/m^2^ (91). If the spectral data are not known, the toolbox might still be used if it can be assumed that the light emission of this phone conforms to the CIE illuminant LED-B3 built-in into the toolbox (however, as will be shown, this assumption is not tenable). On this tentative basis, the melanopic radiance, the melanopic equivalent daylight (D65) luminance (melanopic EDL) and the melanopic photon irradiance can be calculated with the toolbox (see Figure 4A). As the screen subtends an angle of approximately a 5th of a steradian at a viewing distance of 150 mm, the melanopic irradiance, melanopic EDI and melanopic photon irradiance can be obtained as follows:

$$\begin{array}{l} \text{melanopic irradiance }=\text{melanopic radiance }\times \text{ solid angle}\\ \;\approx \;308{\text{mW}/\text{sr}/\text{m}}^{2}\times 0.2\text{sr}=61.6\;{\text{mW}/\text{m}}^{2}\\ \text{melanopic EDI}=\text{melanopic EDL }\times \text{ solid angle}\\ \;\approx \;232{\text{cd}/\text{m}}^{2}\times 0.2\text{sr}=46.4\text{ lx}\\ {\text{log}}_{10}\text{melanopic photon irradiance}/\left({\text{cm}}^{-2}\cdot {\text{s}}^{-1}\right)\\ \;\approx \;13.88+\log_{10}\left(0.2\right)\;\approx \;13.18 \end{array}$$

However, we may not be able to rely on the above estimates. We assumed that the spectrum of the mobile phone conforms to LED-B3. This is likely to cause problems, as the spectrum from mobile phones may have a higher blue content and, unlike LED-B3, is produced by three or more single color LEDs rather than by using a blue LED in combination with a yellow phosphor. In order to replace the above estimates with accurate figures, we need to use the actual spectral data.

#### Spectral Data Approach

When using the toolbox with the spectral irradiance data collected for the selected LED screen [ID 13, (91)], the toolbox output sheet (see Figure 4B) gives the following results:

$$\begin{array}{r} \text{melanopic irradiance }\approx \;85\;{\text{mW}/\text{m}}^{2}\\ \text{melanopic EDI }\approx \;64.3lx\\ {\text{log}}_{10}\text{melanopic photon irradiance}/\left({\text{cm}}^{-2}\cdot {\text{s}}^{-1}\right)\;\approx \;13.32 \end{array}$$

This spectral analysis shows that the simplified approach with the assumption that the phone's light emission conforms to LED-B3 resulted in underestimating the melanopic irradiance and EDI by almost 30%.

Exposure at 150 mm distance from a phone screen (at full white power) is a plausible worst-case scenario for mobile screen use in children and young adults, but it is unlikely that the screen would be used in its brightest setting only. The mix of light and dark within the images displayed on the screen will reduce the spatially-averaged screen brightness as well as the time-averaged melanopic EDI measured at the user's eye. The brightness and the blue emissions may also be reduced in power in the evening using a suitable app. Finally, holding the phone at a further distance reduces the average melanopic EDI incident at the eye, by reducing the “visual” field occupied by the screen.

In preliminary guidance on applying “proper light at the proper time,” and in the absence of a formal consensus, a CIE position statement (59) recently recommended using melanopic EDI as an interim approach to manipulate non-visual responses. Further guidance on this approach is awaited from the participants of the 2nd International Workshop on Circadian and Neurophysiological Photometry (held in Manchester, August 2019), and this is expected to take the form of a scientific publication with melanopic-EDI centered recommendations for healthy indoor light exposures. Further research may be needed to investigate the potential limitations of using melanopic EDI in such recommendations and to explore the correlations between the α-opic quantities and non-visual responses in more detail. While this knowledge develops, and acknowledging the considerations set out in the introduction, the melanopic action spectrum can be considered a good model for predicting melatonin suppression responses: a melanopic EDI below 4 lx results in minimal responses (<25% of maximum melatonin suppression) and a melanopic EDI above 300 lx strongly suppresses salivary melatonin (>75% of the maximum), depending on the exposure duration and experimental context (21). Furthermore, dose-response relationships are subject to a large interindividual variability, for instance the human sensitivity to light for melatonin suppression (i.e., the melanopic EDI needed to produce 50% of maximum melatonin suppression) is reported to vary between individuals by more than one order of magnitude, based on the 95% confidence interval (25). Together with the melanopic EDI values in Table 2, these findings provide inconclusive evidence whether the melatonin suppression induced by mobile phone light emissions in the evening are at levels that raise practical concerns. However, the possibility still remains that prolonged evening use of indoor electric lighting may result in light exposures that are relevant for melatonin suppression.

**Table 2:** Color, RGB, illuminance, scotopic illuminance, and melanopic EDI of a modern mobile phone the screen set to a uniform color at its maximum brightness, and as viewed at a distance of 150 mm [phone ID 13 from (91)].

**Screen color**	**RGB values**	**(Photopic) illuminance, lx**	**Scotopic illuminance, lx**	**Melanopic EDI, lx**
1. White	255, 255, 255	73.3	164.6	64.3
2. Purple	48, 0, 179	3.1	30.2	15.5
3. Blue	0, 0, 210	3.6	42.6	22.0
4. Green	0, 100, 100	7.8	21.8	8.6
5. Lilac	102, 0, 255	7.8	65.9	34.0
6. Cyan	142, 201, 230	40.4	110.8	45.0

Furthermore, whilst the studies mentioned above suggested that mobile phone screens can have statistically significant effects on sleep, a more representative comparison (99) demonstrated that a 4-h exposure to an e-reader compared to a printed book (when repeated on five consecutive nights with a scheduled 06:00 am morning wake-up time) only resulted in an average reduction of 5 min in total nightly sleep duration and 12 min in REM sleep duration, so these effects of light may be less significant in a practical sense. Insufficient exposure to light during the day in modern (indoor) lifestyles may be of greater concern, and, as set out earlier, daytime light exposures increase the robustness of circadian rhythms and reduce the disruption caused by light exposures in the evening, see the Introduction section and Figure 1D.

## Concluding Remarks

Daily variations in the light environment are important for sleep, well-being and long-term health. The knowledge base concerning the contributions and interactions of retinal photoreceptors in driving non-visual effects is becoming more mature. Although the science is by no means complete, measures of the environment expressed in terms of melanopic EDI are now thought to have ecological validity. New recommendations for future building and lighting standards are therefore expected to incorporate both minimum thresholds for daytime melanopic EDI and maximum thresholds for evening melanopic EDI. These recommendations should be carefully integrated with the visual components within existing lighting codes. One way of limiting evening melanopic EDI would be by recommending dimmer lighting, and this is more effective when simultaneously lowering melanopic DERs (i.e., reducing M/P ratios). Another recommendation could be to strive for near darkness wherever people are expected to sleep at night. The CIE S 026 Toolbox has been introduced, partly to support this expected shift in lighting practice, and partly to enable researchers to expand the evidence base for future lighting standards, guidance and health advice.

Figure 5D shows that the melanopic DER for daylight on a clear day is significantly greater than the melanopic DER within a recent sample of white LED lighting with a range of CCTs. This supports the viewpoint that the LEDs sampled are relatively inefficient at producing melanopic light for a given combination of CCT and luminous flux, in agreement with others (97, 98). New lighting products, including those with tunable M/P ratios, may help to address this. Higher M/P ratios, similar to daylight, might be considered a beneficial characteristic for the daytime indoor environment. Daylight entry within the built environment is a good way to achieve this.

If the aim is to minimize melanopic light exposures, the lighting used at night for navigation and perceptions of safety should be restricted to lower M/P ratios. Increased daytime light exposures can reduce the adverse effects of evening light (39–46), and daytime light exposure may be as important as avoiding bright light before bedtime. During the day, indoor electric lighting could reproduce the melanopic light exposures (and other facets) of the outdoor environment, although this entails greatly increased indoor illuminances. Nevertheless, daylight is an excellent, natural, energy-efficient source of melanopic-rich light, and public health policies should encourage a daytime (natural) light-seeking lifestyle, especially during the first morning hours after bed and starting from the very first days after birth.

## Data Availability Statement

The raw data supporting the conclusions of this article are subject to UK Crown copyright and will usually be made available by the authors, without undue reservation.

## Author Contributions

All authors listed have made a substantial, direct and intellectual contribution to the work, and approved it for publication. Both authors have contributed extensively to the gray literature on this topic in unpaid voluntary roles, including (12, 59, 89, 90).

## Conflict of Interest

LS's full time position at Eindhoven University of Technology is partially funded by Signify. The remaining author declares that the research was conducted in the absence of any commercial or financial relationships that could be construed as a potential conflict of interest.

## Footnotes

^1^For readability, 555 nm will be written instead of λ_d_ ≈ 555.016 nm for the wavelength of light corresponding to a frequency of 540 × 10^12^ Hz for light in standard air.

^2^D65 represents daylight with a color temperature of ~6,500 K. Other reference illuminants (like standard illuminant A or equi-energy illuminant E) could be used instead of D65 to define equivalent illuminances, but such non-standard quantities should be avoided as much as possible. Lucas et al. (13) adopted the equi-energy illuminant (E) as the reference illuminant when introducing the “α-opic equivalent illuminance” concept for non-visual metrology (13, 89), but without explicit mention of the reference illuminant selected.

^3^In the same order, the photometric quantities that correspond to this list are α-opic equivalent daylight (D65) light exposure [lx·h], α-opic equivalent daylight (D65) luminous energy [lm·s], and α-opic equivalent daylight (D65) luminous intensity [cd].

^4^The energy, *E*, of a single photon depends on Planck's constant, *h*, the speed of light, *c*, its wavelength, λ, and the refractive index of the medium (*n*_air_, say), so that for *N*_p_ photons (as it is expressed in the photon system) at a given wavelength, the corresponding radiometric energy is *E*_e_ = *N*_p_·*h*·*c*/λ·*n*_air._ When converting action spectra from the photon system to the radiometric energy system, or vice versa, once the adjustment has been applied at every wavelength, the entire action spectrum must also be renormalized so that its new maximum value is equal to 1.
